# Computer-Based Prognostic Task Measurements as Indicators of Uncertainty Acceptance

**DOI:** 10.3390/ejihpe10010016

**Published:** 2019-11-11

**Authors:** Vitalii Epishin, Nataliya Bogacheva

**Affiliations:** Clinical Psychology Department, I.M. Sechenov First Moscow State Medical University (Sechenov University), Moscow 119991, Russia; v.e.epishin@gmail.com

**Keywords:** prognostic task, decision-making, tolerance for uncertainty, intolerance for uncertainty

## Abstract

There were few studies of individual differences in prognostic decision-making from the psychological point of view; most of them focused on the differences between novices and experts making the prognoses. In this study, we suggested a new task that matched the criteria of a prognostic one, was computerized, and did not require expertise in any field of knowledge. Thus, the proposed method investigated how people processed information and controlled uncertainty in prognostic tasks. On a sample of 78 people aged 17–66, we used a quasi-experimental design to find the patterns of the proposed task parameters and how they correlated with personality and cognitive variables. Five well-known personality questionnaires accessing traits, known to be included in decision-making regulation, were used along with a cognitive abilities test to measure those variables. Two patterns were identified via cluster analysis. Differences in intolerance for uncertainty were demonstrated for the people from two identified clusters. Those patterns could be interpreted as uncertainty control strategies for decision-making grounding in prognostic tasks.

## 1. Introduction

Most of the studies of forecasting and prediction in psychology over the past decades were somehow related to cognitive biases and heuristics, leading to incorrect estimation of the probabilities of certain events. That research area originated from the works of Kahneman, Slovik, and Tversky [1,2]. According to most studies, intuitive forecasting accuracy was significantly less accurate than statistical methods and thus required corrective procedures to increase its efficacy [3,4]. Numerous studies showed how the form of data presentation and the type of data influenced the accuracy of forecasts [5,6]. The role of feedback in improving the forecasts’ accuracy showed mixed results, depending on the type of feedback [7,8]. Numerous works were also devoted to the comparison of forecasts performed by beginners and experts in different fields [9,10,11]. The initial understanding of judgment as a source of lower forecast accuracy was subsequently revisited [12], suggesting that the research should concentrate more on human judgment support in various areas.

At the same time, relatively little attention was paid to the problem of individual differences and the role of personality variables in forecasting and prognosis. Wright and Ayton showed a significant contribution of individuals’ characteristics to judgmental forecasting [13]. The authors concluded that people differ significantly in their prognostic abilities due to their personality variables, while situational factors were non-significant for the forecasting. Another study showed that intelligence and cognitive styles were significant predictors of the accuracy of geopolitical forecasts [14] with cognitive flexibility and open-mindedness being the key factors of successful predictions (along with specific skills in probabilistic reasoning and general knowledge in politics). The role of intelligence in forecasting success was also demonstrated in a study using the Iowa Gambling Task, although this task can only relatively be considered prognostic [15].

Among personality traits that mediate decision-making in uncertain situations, the key variables were those that reflect personal attitudes towards uncertainty. Frenkel-Brunswick was one of the first researchers to use the terms tolerance and intolerance for uncertainty [16]. She showed that those traits affected both cognitive and emotional domains. Numerous studies demonstrated correlations between tolerance and intolerance for uncertainty and stable personal, cognitive, and emotional traits. In this aspect Furnham and Marks argued whether it was possible to consider tolerance and intolerance for uncertainty as second- or third-order factors to personality traits, for example, the Big Five traits [17].

Among other personality factors that could mediate decision-making in uncertain situations were impulsiveness [18], intuition [19], risk-readiness, and rationality [20]. Uncertainty coping strategies also played an important role in decision-making [21]. In the current study, we tried to assess the relationships between all the above-mentioned factors and the strategies for uncertainty control in a specially designed prognostic task.

In most studies, tasks from a certain domain of interest were used as prognostic tasks. Among them were sales forecasting [22], sports results [10], election results [23], geopolitical events [14], weather [24], and so on. This approach could not entirely exclude the influence of personal knowledge and experience in the domain, thus making it difficult to assess the dispositional variables contribution to the forecast.

Another common trait of the existing research was the use of the tasks in which a person had either to extrapolate time series or to assess the probability of an event according to the data available on the probabilities of possible outcomes. In our opinion, this might determine certain results. E.g., forecasting specialists were more used to or at least acquainted with this type of data presentation while non-specialists could have a very different experience with those types of information. Thus, such tasks were better suited for the study of cognitive biases and limitations of the human cognitive system in making probabilistic judgments. Yet, in real life, a deciding human (not a specialist) would deal with sequences of events, and not with their formal representation via time series, trends, probabilistic estimates, etc. [5].

On this basis, one of the aims of this study was to develop and test a model of a prognostic task, which, on the one hand, would to the limit exclude the use of domain-specific experience and knowledge when making a prognosis, and on the other hand, would avoid the use of a formal data presentation and instead be more a process, a series of events for which the participant would have to make a prognosis.

To define a task as prognostic, it must meet several criteria. Regush [25] suggested the following characteristics: (1) the goal of the task was to build knowledge about the future; (2) the condition of the task did not contain sufficient data to perform a prediction; (3) the relationship between the given and the sought was probabilistic; (4) the direction of the search in the task was not specified by the conditions of the task. Thus, an inherent characteristic for a prognostic task was uncertainty, either uncertain conditions or subjective uncertainty. Both situational and dispositional factors could be the sources of the latter. Situational factors manifested as situational limitations forcing a person to make decisions with an incomplete orientation in the context. Dispositional factors were the subject’s characteristics, including personality (for example, motivation, accessibility of internal experience, etc.) and cognitive traits (intelligence level, executive functions, etc.).

In the computerized task that we proposed as an experimental model of the prognostic situation, the participants were able to choose the moment when they were ready to make a prognosis independently, thereby adjusting the acceptable level of uncertainty in the situation (see Section 2.2.1 for more details). The parameters defining the conditions of uncertainty were: (1) the ratio of available and missing information (awareness); and (2) how strongly the available information confirmed one of the alternatives (justifiability). There was also a parameter that had little real prediction value yet could be seen as such by the participants—(3) the temporary tendency in the appearance of the new bits of information (trend). The participants were motivated to decide as soon as possible, with a minimally acceptable level of uncertainty, by a payment matrix setting up the value of the prognosis. Two interconnected parameters determined this value (same as in most real-life prognostic situations): timeliness and accuracy. Improving the accuracy and reasonability of the prognosis implied a detailed orientation, requiring more information and time (thus, timeliness decreased when accuracy increased).

Anticipation of possible outcomes and their probabilities, information gathering, hypothesizing, and analyzing the hypotheses are the essence of human prognostic activity, as they reduce the subjective uncertainty of the situation. Thus, choosing a decision-making moment associated with a certain forecast value (the value of a possible gain), one chose a subjectively acceptable level of uncertainty.

Along with testing the proposed task, our study aimed to assess the regulatory role of personality traits (rationality, risk-readiness, impulsiveness, tolerance, and intolerance for uncertainty, intuitive ability, engagement, and uncertainty coping strategies) as well as cognitive abilities in solving prognostic tasks. We supposed that those would manifest through the preference for different levels of the prognosis value and different strategies of mastering the task. Based on that notion, several research hypotheses were formulated:

1. Awareness (a more complete orientation in the situation before decision-making) was positively associated with rationality and intolerance for uncertainty.
2. The prognosis justifiability as the reliability of the possible outcome derived from the information available, positively correlated with the level of intelligence and negatively—with the risk-readiness and venturesome-ness.
3. The trend, reflecting the tendency to react to momentary changes in a situation when giving a prognosis, was positively associated with impulsiveness and unproductive coping, hypervigilance, manifested as a tendency to make impulsive decisions in an uncertain situation.
4. There were stable correlational patterns of awareness, justifiability, and trend, which indicate strategies for uncertainty control were associated with differences in personal and cognitive traits involved in decision-making regulation.

## 2. Materials and Methods 

### 2.1. Participants

The voluntary participants for the research were recruited via advertising in the students’ groups (Clinical Psychology Department of Sechenov University) and the “snowball” sampling afterward. As we were aiming to assess the capabilities of the new game task, the inclusion criteria were rather broad: Russian as the native language to ensure understanding instructions, age over 17 years old, and completed secondary education. Initially, 105 people were recruited. In further processing, partial protocols were excluded, and only full protocols with all tasks done and no signs of random answers were analyzed, leaving us with 78 participants. Among that sample, there were 64 women and 14 men, aged 17–66 (М = 24; SD = 10, Me = 21), including 60 undergraduate students and 18 participants with a university degree.

### 2.2. Methods and Outcome Measures

#### 2.2.1. Prognostic Task

**Figure 1:**
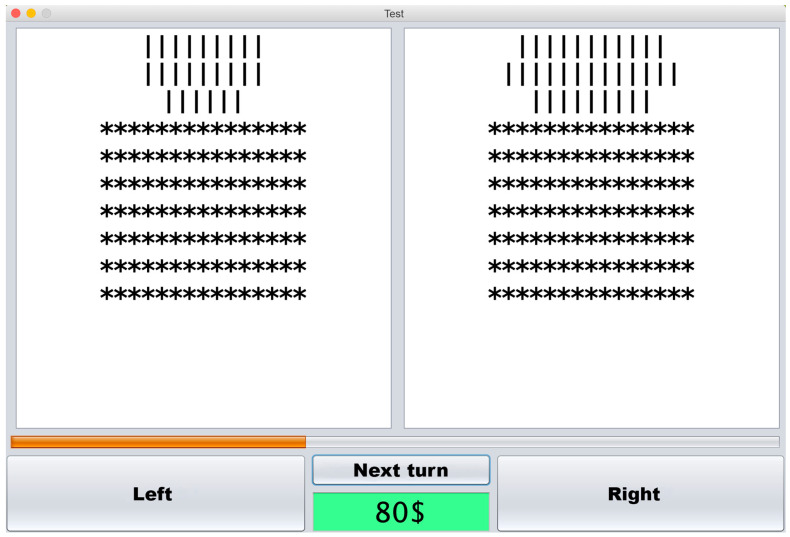
Prognostic task interface. With “left” and “right” buttons the participant could make the final decision about the part of the screen with most vertical lines, “next turn” button revealed the next row of vertical lines (masked by “*” symbols) that was also revealed automatically seven seconds after the previous one. The green square indicates the amount of the in-game currency left.

Computerized modification of Azarov’s [26,27] cognitive decision threshold-measuring task was developed to serve as a prognostic task for our research. In this modification, the task was presented on a monitor screen, which was divided vertically into two halves. On both parts of the screen, 10 identical rows, consisting of “*” symbols were displayed. At the beginning of the task, the first rows were opened on both halves of the screen simultaneously, revealing sets of vertical lines, different in both halves of the screen. Differences in the number of lines between the left and right parts of the screen could vary from 0 to 5 at each step of the task. The first row was displayed for 7 s, after which the second row would open, and so on. The time interval between the opening of the rows was enough for the participant to see the whole picture but too short to count the exact amount of the lines, so the decision had to be made on the general impression. The participant’s goal was to predict which half of the screen would contain more vertical lines after all rows were opened. The selection was made by pressing the button at the bottom of the corresponding half of the screen (See Figure 1).

The game used a payment matrix to encourage the participants to make their choice as early as possible: at the beginning of each series, each participant received 100$ (in-game currency with no real-life money gains or losses involved). For each row opened, 10$ were deducted. If the participant made a wrong choice (chose the part of the screen which ended up with a lesser total amount of lines) (s)he was fined 50$. Thus, to maximize the final gain, the participant had to make a choice as early as possible, but at the same time, try not to make mistakes. In order to do that (s)he had to rely on the situation present at the time of the decision-making. The whole task consisted of 30 trials.

The game scenario was the same for all participants. Before the main task, the participants were asked to go through a training session to get acquainted with the program and the rules.

In the original Azarov’s task, the only measurement used (as the “cognitive decision threshold”) was the average number of moves (average number of rows opened before making the decision) in all the trials. We used three measurements instead. While solving the prognostic task, the participant could rely on two parameters of the current situation: (1) the ratio between the opened and closed rows, and (2) the difference in the number of lines in opened rows between the left and right halves of the screen. With more rows opened (higher indicator 1) and a more noticeable difference between the left and right halves of the screen (higher indicator 2), the likelihood of the correct prognosis was higher. At the same time, according to the payment matrix, the gain would be less with each additional row opened. Thus, we suggested that those indicators specify an individually acceptable measure of uncertainty at which the participant was ready to make his/her prognosis. We designated those two indicators as awareness and justifiability, respectively. We also deduced the third parameter for the participants to use in their decision-making, which we called trend (considering the direction, it could be either negative or positive). This indicator was not intended from the beginning of the research but rather found during the data gathering and evaluation process. It was noticed that the participants were often making their decisions at the very moment when a newly opened row of vertical lines gave a noticeable boost to the general difference between the number of lines on the right and on the left. Those changes were not supposed to be sustainable, yet the participants seemed to react to them as if they were. The trend indicated the way the number of lines in the two halves of the screen was increasing or decreasing compared to the previous step. The previous studies also supported the idea to include the trend as one of the possible indicators, showing how non-experts’ prognoses changed in situations of upgrade and downgrade trends previously shown in the experts’ prognoses in that field [28,29].

The above-mentioned parameters were gathered automatically by the program used to operate the prognostic task.

#### 2.2.2. Psychodiagnostic Methods

- Personal Decision-Making Factors Questionnaire (LFR-21) developed by Kornilova [30]. The questionnaire consisted of 21 yes-or-no statements divided into two scales: (1) rationality, an inclination for a broader orientation in the decision situation, and (2) personal risk-readiness, willingness and ability to make and perform choices in uncertain situations.
- New questionnaire of tolerance/intolerance for uncertainty (NTN) developed by Kornilova [30]. The questionnaire consisted of 33 statements, evaluated with a seven-point Likert scale (from “completely disagree” to “completely agree”). The questionnaire provided three measurements: (1) tolerance for uncertainty, a personal acceptance of novelty, complexity, inconsistency problem-solving and decision-making conditions; willingness to act in new and unusual ways; (2) intolerance for uncertainty the desire for clarity, orderliness, avoidance of uncertainty, reliance on rules and principles; a tendency to see opinions, values and methods of action as either right or wrong; (3) interpersonal intolerance for uncertainty as a desire for control in interpersonal relationships, a desire for clarity and discomfort in relationships where this clarity is absent.
- Eysencks’ Impulsiveness Scale (seventh version, I7) [31] in Russian short adaptation by Kornilova and Dolnikova [32]. Consisted of 28 yes-or-no questions and three sub-scales: (1) Impulsiveness, a decrease in self-control and a tendency to act under the spur of the moment; (2) Venturesomeness which manifested in the search for strong emotions, thrills; (3) Empathy, reflecting the ability to empathize with others and feel their emotions.
- Russian version of Epstein’s Faith in Intuition scale from the Rational Experiential Inventory (REI) [33,34] with two measurements: (1) experiential (intuitive) ability—The ability to report one’s intuitive impressions and feelings, and (2) experiential (intuitive) engagement as a willingness to make decisions depending on intuitions and feelings.
- Melbourne decision making questionnaire (MDMQ), a Russian adaptation by Kornilova [35,36]. The Russian version retained a 22-statement structure with four scales: (1) vigilance—A productive uncertainty coping strategy, the desire to carefully consider possible alternatives for the decision-making; and three unproductive copings: (2) buck-passing—The desire to abandon independent decision-making; (3) procrastination—The desire to delay decision-making; and (4) hypervigilance—The tendency to decide impulsively, the desire to get rid of the uncertain situation without intellectual orientation in it.
- Brief screening test (BST), the Russian modification of The Wonderlic Personnel Test. The test consisted of 50 questions. For the Russian version, participants had 15 min to complete as many questions as possible to assess their level of general cognitive abilities [37].

### 2.3. Statistical Methods

K-means clustering was used to identify the stable patterns of prognostic task measurements. Cronbach’s Alpha coefficient was applied to evaluate the internal consistency of the strategy indicators. For group comparison and correlational analysis, non-parametric methods were chosen due to sample-sizes and non-normal data distribution. Thus, Mann–Whitney U test and Spearman’s rank correlation coefficient (rho) were used for further calculations. All calculations were made in IBM SPSS Statistics version 22. 

## 3. Results

### 3.1. Internal Consistency of the Prognostic Task Performance Indicators

First, we wanted to see whether the performance indicators in our prognostic task (described in Section 2.2.1.) could be considered stable individual decision-making characteristics. To do that the evaluation of their internal consistency was required, and we used Cronbach’s Alpha for that. The obtained values of the coefficient were: for awareness α = 0.987, for justifiability α = 0.924 and for trend α = 0.555. Awareness and justifiability together set the level of uncertainty of the situation at the time of the decision-making. With lower values of each of them, the risk of a wrong prognosis increased. Such high Cronbach’s Alpha values for those indicators reflected the stability of individual preferences for forecast value, determined by the risk ratio (as an unacceptable level of uncertainty) and potential gain. Trend was found the least internally consistent of the three, possibly meaning that it was to a larger extent related to situational, rather than stable factors of decision-making.

### 3.2. The Relationship between Psychological Measurements and the Prognostic Task Performance Characteristics

Only a few significant correlations were found for the prognostic task indicators (see Table 1).

Two main indicators of the prognostic task performance (awareness and justifiability) showed significant positive correlations with cognitive abilities. Together, those two indicators defined the uncertainty of the current in-game situation. Thus, the level of cognitive abilities was associated with the tendency to make more reliable prognosis, based on the current situation, even though the potential gain was reduced.

The trend indicator only positively correlated with the tolerance for uncertainty scale (NTN). 

### 3.3. Identification of Uncertainty Control Strategies in the Prognostic Task Solving

We supposed that there were stable patterns of awareness, justifiability, and trend which the participants preferred when they needed to determine the uncertainty of the game situation. To further identify those patterns, a K-means cluster analysis was carried out using the K-means method. As variables for clustering, all three game strategy indicators were used. We considered two-, three- and four-cluster solutions. The convergence with a small number of iterations (4) was achieved for a two-cluster solution (see Table 2).

Participants from the first cluster preferred to make more risky prognosis, making a choice when fewer rows were opened (lower awareness) and with a smaller difference in the number of vertical lines between two halves of the screen (lower justifiability), but at the same time they relied more on the situational cumulative difference change after the last row opened (higher trend), compared to the participants from the second cluster.

Then we assessed the internal consistency of the awareness, justifiability, and trend indicators separately in each cluster using Cronbach’s Alpha coefficient (see Table 3).

As shown in Table 3, the Cronbach’s Alpha coefficient values differed for the first and second clusters. In both groups, awareness (the ratio of the number of opened and closed rows at the time of the decision-making) demonstrated high reliability. For justifiability (the difference in the number of lines in the two halves of the screen at the time of the decision-making), a sufficient level of internal consistency was observed only for the participants from the first cluster. According to the trend (row-wise change in the number of lines in two halves of the screen at the time of the decision-making), Cronbach’s Alpha did not reach satisfactory levels in both clusters. This might mean that for the participants in the first cluster, awareness and justifiability were stable characteristics of acceptable uncertainty control strategies in the decision-making. For the participants in the second cluster, only awareness was found to be a stable characteristic.

Spearman’s rho coefficients were calculated to see if the values of the performance indicators correlated in both clusters, (see Table 4).

While awareness and justifiability showed similar strong positive correlations in both clustered groups, the relationships of those two parameters with the third parameter—trend—were different for both clusters. The negative correlation between trend and both awareness and justifiability indicated that the participants from the first cluster were making their prognoses earlier and with a smaller observable difference in the number of vertical lines between the left and right parts of the screen, at least when there was a noticeable change in that difference after the next row was opened (higher trend). In the second cluster, those correlations were both insignificant (and the correlation between awareness and trend was almost zero). This meant that the participants from this group did not rely on the momentary changes between the newly opened rows while evaluating the game situation to make their prognoses.

### 3.4. Personality Specifics of the Participants from Different Clusters

Mann-Whitney criterion was used to compare personality specifics and cognitive abilities in both clustered groups, as most of the variables were not normally distributed. Significant differences (U = 558.0, *p* = 0.047) were found for only one scale—intolerance for uncertainty (NTN questionnaire). The mean score for the first cluster was 59.38 comparing to 55.47 in the second cluster. Thus, the participants from the first cluster were significantly more uncertainty intolerant.

## 4. Discussion

We rejected our first hypothesis on awareness in the prognosis task being related to personality traits, crucial for the decision-making. On the contrary, both strategy indicators, awareness and justifiability, showed no direct relationship with personality traits. Yet, the relationship between justifiability and general cognitive abilities level allowed us to accept the second hypothesis partially. Besides, cognitive abilities were found positively correlated with awareness, making both strategy indicators linked with general cognitive abilities. Previous studies demonstrated the role of intelligence and cognitive abilities in general in the regulation of choice strategies when solving prognostic tasks (e.g. the Iowa Gambling Task) [15]. The part of the second hypothesis about personality traits relating to the justifiability in the prognostic task was rejected. The absence of significant relationships between the measured dispositional traits and the preferred riskiness of the forecasts could be explained, on the one hand, by the relatively low contribution of dispositional variables to real behavior (explaining not more than 20% of the variance) [38]. Another possible explanation was related to the interaction of many personal factors. That interaction of factors was not evaluated in this study due to the small sample size. The latter explanation seemed to be preferable because awareness and justifiability showed high internal consistency.

While there were no other significant correlations between awareness, justifiability, and personality traits, a positive correlation was found between the decision strategy indicator trend and intolerance for uncertainty. That is, people with high intolerance to uncertainty trait relied more on the current game situation changes while making a prognosis. That allowed us to reformulate our previous assumption about the personality regulation factors manifested in the trend strategy indicator. We had to reject hypothesis 3 about the trend’s relationship with impulsiveness and risk-readiness. However, we could suggest that intolerance for uncertainty should be included in the participant’s control over the dynamics of changes in situational uncertainty due to established strong positive correlations.

It should be noted that trend (unlike awareness and justifiability) did not predict the outcome—This was not assumed by the game scenario and was not in the game code. Thus it could be seen as a false indicator making decisions more difficult for the participants. The obtained result confirms the data on the relationship between irrational gambling beliefs and intolerance for uncertainty [39].

The results obtained in our study indicated that two of the three strategy indicators in the prognostic task (awareness and justifiability) demonstrated high internal consistency, and, therefore, can be considered as stable individual decision-making criteria for at least this particular task. We considered their levels as a comparative measure of a subjectively acceptable level of uncertainty (the higher their level, the less uncertainty is left in the decision-making). The established correlations of those two parameters with the cognitive abilities might indicate that the regulatory role of cognitive components of the intellectual and personal potential was manifested to a greater extent [40]. A possible explanation for the insignificant relationship between awareness and justifiability with personality traits could be that behavioral acceptance of risk and uncertainty was to a larger extent determined by situational rather than dispositional factors, in which the relationship with risk and uncertainty was reflected but not actively used in practice [40,41].

The results allowed us to accept the 4th hypothesis. Two identifiable patterns of awareness, justifiability, and trend were found, indicating two distinct strategies for uncertainty control in the prognostic task solving. The first strategy involved a steady reliance on most parameters of the situation and their relationship (evidenced by the high Cronbach’s Alpha values for awareness and justifiability and their significant correlation with the third parameter—trend). The use of this strategy in our task turned out to be connected with the tendency to make riskier forecasts. At the same time, the participants, who used this strategy showed higher levels of intolerance for uncertainty. We suggested that the desire for maximum clarity, characteristic of individuals with high rates of intolerance for uncertainty, in this situation, could be associated with the search for as many guidelines to reduce the uncertainty of the situation as possible. Yet, in this task, it was not the best strategy, according to the results. In our study, the average number of correct predictions for people using the first strategy was 17.24 (which is slightly better than the probability of random guessing in 30 trials) versus 20.67 for those who preferred an alternative, second strategy. The second strategy was associated with a stable reliance on one indicator—awareness (only that variable showed a satisfactory value of Cronbach’s Alpha). People who used this strategy preferred to make a prognosis based on the ratio of available and missing information, opening more rows in the task to secure their decision.

Several limitations should be mentioned. The small sample size limited the ability to summarize the results of our research. Another important limiting factor was the use of the only payment matrix. Using several different payment matrices would be necessary to generalize the results further and to see whether the strategies would be stable or dynamic if the lose-gain ratio were different. The large dispersion of the participants’ age and heterogeneity of its distribution in the sample did not allow us to assess whether the choice of strategies and correlations between the variables were related to this factor. This might be the goal for further research.

## 5. Conclusions

1. The results indicated that the characteristics of a prediction-based situation (ratio of available and missing information and justifiability of a possible choice) could be considered as individually stable subjective guidelines when making decisions in prognostic tasks. At the same time, the absence of significant relationships with the measured dispositional characteristics left open the question of their contribution to the regulation of human prognostic activity and would require further research.
2. The level of general cognitive abilities was associated with the tendency to make more informed prognoses based on more information available.
3. Two strategies for decision-making in prognostic tasks were identified, differing in the reliance on the available information. The first strategy involved more indicators to rely on, yet it was less successful than the second strategy, characterized by sustainable reliance only on the awareness parameter. The first strategy was related to the tendency to give earlier and less informed prognoses.
4. Intolerance for uncertainty was associated with a more detailed orientation in the task, manifesting in the tendency to take more parameters into the account, yet, without a full understanding of their real contribution to the final result, which could lead to an underestimation of the role of significant parameters, and made the prognosis more risky and less justified. These findings allowed us to look from a new perspective on the connection of intolerance for uncertainty and various irrational beliefs. Intolerance for uncertainty could manifest in the tendency to search for patterns in random events and the uncritical adoption of such patterns as the basis for decision-making.

## Figures and Tables

**Table 1:** Significant correlations (Spearman’s rho) between awareness, justifiability, and trend in the prognostic task and psychodiagnostic characteristics.

	Awareness	Justifiability	Trend
**Cognitive abilities (BST)**	0.261	0.244	
**Tolerance for uncertainty (NTN)**			0.226

All significant with *p* < 0.05.

**Table 2:** Final cluster centers, N indicates the number of participants in each cluster.

	Cluster 1 (N = 42)	Cluster 2 (N = 36)
**Awareness**	2.10	4.27
**Justifiability**	4.10	6.52
**Trend**	2.03	1.63

**Table 3:** Internal consistency (Cronbach’s Alpha) of the performance indicators of clustered groups.

	Cluster 1	Cluster 2
**Awareness**	0.962	0.958
**Justifiability**	0.825	0.606
**Trend**	0.468	0.405

**Table 4:** Correlations between prognostic task strategy indicators in two groups.

	2nd cluster	Awareness		Justifiability		Trend	
1st cluster							
***Awareness***				0.926 ** (0.981 **)		−0.004 (−0.403 **)	
	
***Justifiability***		0.930 ** (0.981 **)				0.273 (−0.318 **)	
	
***Trend***		−0.565 ** (−0.403 **)		−0.459 ** (−0.318 **)			
	

** is significant with *p* < 0.01. The results for the first cluster are below the diagonal line, for the second cluster, they are above the line. Correlation coefficients for the entire sample are in parentheses.
